# Supplementary material for: Suicide Overall and Suicide by Pesticide Rates among South Korean Workers: A 15-Year Population-Based Study
Source: Int J Environ Res Public Health. 2019 Dec 3;16(23):4866. doi: 10.3390/ijerph16234866 (PMC6926852; doi:10.3390/ijerph16234866)
Supplement: Supplementary file 1 [file ijerph-16-04866-s001.pdf]

Supplementary Table S1. The number of suicide deaths (X60-X84 and Y87.0) from the National Death Records.

Year	MNP		OST		AFF		SKL		USL	
	Male	Female	Male	Female	Male	Female	Male	Female	Male	Female
2003	83	19	1084	239	632	133	637	48	351	25
2004	94	31	1125	228	617	127	626	27	371	29
2005	127	34	1087	337	550	114	588	33	313	29
2006	101	27	1011	238	445	92	487	28	261	14
2007	117	62	1029	413	438	83	491	70	230	38
2008	322	121	1075	484	377	82	410	28	301	37
2009	526	189	1465	586	385	74	479	25	394	36
2010	585	168	1530	518	399	69	447	18	354	33
2011	529	161	1613	478	397	66	523	16	387	30
2012	598	129	1387	445	330	57	518	26	487	62
2013	768	164	1432	444	333	46	616	22	599	69
2014	757	172	1443	440	251	34	580	36	674	66
2015	728	137	1276	425	242	33	608	29	631	50
2016	762	149	1197	452	218	22	555	29	634	56
2017	727	134	1159	414	205	24	513	17	555	55

The number of deaths where the population is assumed to be 1,000,000. **MNP:** Manager-Professional, **OST:** Officer-Service-Trade, **AFF:** Agriculture-Forestry-Fishery, **SKL:** Skilled manual labor, **USL:** Unskilled manual labor.

Supplementary Table S2. The number of suicide by pesticide deaths (X68) from the National Death Records.

Year	MNP		OST		AFF		SKL		USL	
	Male	Female	Male	Female	Male	Female	Male	Female	Male	Female
2003	14	5	260	102	373	83	159	16	116	10
2004	11	4	262	70	395	95	160	9	113	12
2005	17	2	183	47	334	81	127	6	70	7
2006	13	2	152	38	267	65	88	2	75	3
2007	15	0	151	34	286	56	92	5	50	10
2008	33	6	139	52	220	58	65	5	67	11
2009	55	8	138	43	226	51	61	2	73	13
2010	38	6	122	35	214	42	52	2	77	1
2011	47	7	122	38	206	47	51	4	68	6
2012	30	3	86	20	156	38	38	1	55	14
2013	27	3	43	10	119	22	30	1	45	4
2014	16	2	33	7	75	15	25	0	41	3
2015	14	0	23	12	53	8	25	1	27	1
2016	14	0	21	13	49	6	14	0	40	2
2017	11	3	21	11	32	8	14	0	23	3

The number of deaths where the population is assumed to be 1,000,000. **MNP:** Manager-Professional, **OST:** Officer-Service-Trade, **AFF:** Agriculture-Forestry-Fishery, **SKL:** Skilled manual labor, **USL:** Unskilled manual labor.

Supplementary Table S3. The population of occupational groups by year from the Economically Active Population Survey over the 15-year period.

Year	MNP		OST		AFF		SKL		USL	
	Male	Female	Male	Female	Male	Female	Male	Female	Male	Female
2003	1416366	758807	3736911	4603379	673080	538538	5323204	1345619	1345619	931470
2004	1402319	764276	3604332	4745807	612265	481796	5578237	1388949	1388949	953944
2005	1427875	835402	3564362	4824317	574224	442298	5634970	1366142	1366142	957545
2006	1485375	893099	3533974	4847189	543584	418502	5744805	1443863	1443863	999205
2007	1536126	909955	3511449	4813705	514680	397462	5828770	1457291	1457291	1028269
2008	2892005	1881010	4040574	4834054	519899	373508	4120572	657398	657398	1309338
2009	2862506	1945590	4136313	4711741	518592	348941	4003530	596796	596796	1356741
2010	2959296	2006115	4082339	4756056	512236	311105	4152483	632603	632603	1383719
2011	2953249	2080690	4274737	4795345	491427	309958	4222596	638798	638798	1414997
2012	2949986	2142967	4435744	4905671	491964	293775	4310774	662441	662441	1368808
2013	2943066	2259586	4546437	4906101	473743	299599	4352094	670038	670038	1359967
2014	3001838	2356167	4640935	5092370	450309	275499	4430609	670709	670709	1337800
2015	2938253	2416791	4615835	4849419	391237	123747	4549522	649861	649861	1346261
2016	2971747	2458347	4643951	4925424	370040	96734	4565729	644122	644122	1326425
2017	2970653	2558240	4694370	5002555	386429	81976	4529558	647376	647376	1296265

MNP: Manager-Professional, **OST:** Officer-Service-Trade, **AFF:** Agriculture-Forestry-Fishery, **SKL:** Skilled manual labor, USL**:** Unskilled manual labor.

Supplementary Table S4. The crude suicide and suicide by pesticide crude rates of the population age 15-64 by sex, including workers and non-workers, per 1,000,000.

Year[1]	Suicide Rate		Suicide by Pesticide Rate	
	Male	Female	Male	Female
2003	303.1	103.1	90.2	41.5
2004	309.2	103.6	93.5	38.7
2005	310.8	128.7	76.5	31.0
2006	276.4	100.5	61.4	25.9
2007	286.9	150.3	62.0	23.6
2008	300.5	155.2	56.5	23.0
2009	374.7	183.3	52.7	21.3
2010	380.3	171.7	50.7	17.1
2011	402.9	159.2	46.5	19.5
2012	354.3	134.1	35.1	14.7
2013	350.8	129.2	21.1	7.9
2014	346.3	126.1	15.7	5.0
2015	321.9	111.4	12.9	4.0
2016	312.7	117.0	12.1	4.5
2017	301.7	108.8	9.5	4.5

Supplementary Table S5. The suicide overall crude rates of the population age 15-64 by age, including workers and non-workers, per 1,000,000.

Year	Suicide Rate									
	15-19	20-24	25-29	30-34	35-39	40-44	45-49	50-54	55-59	60-64
2003	47.7	104.6	147.3	186.8	214.5	249.8	232.5	263.0	308.1	407.1
2004	33.5	91.5	126.1	155.3	214.7	256.4	267.6	296.6	346.3	440.5
2005	46.1	130.3	171.8	189.2	211.0	235.7	283.5	307.1	319.5	454.8
2006	34.2	84.4	136.2	131.1	175.6	202.0	257.1	307.5	326.1	396.0
2007	46.4	131.9	213.8	198.2	201.1	222.2	272.5	305.4	325.2	394.9
2008	41.0	158.7	245.8	205.7	258.6	237.0	286.3	255.7	288.4	331.8
2009	56.1	164.0	284.0	286.8	300.1	287.6	332.4	361.8	340.1	411.5
2010	40.1	145.6	266.2	273.6	278.8	299.2	347.1	357.8	379.2	415.6
2011	45.1	137.5	257.7	275.2	278.6	310.3	319.6	391.0	414.2	442.7
2012	38.1	107.0	187.1	256.5	227.6	286.6	290.4	340.2	373.0	389.1
2013	39.4	94.9	172.4	257.4	258.0	296.6	287.8	345.7	300.7	309.4
2014	42.6	94.3	180.1	243.2	247.3	294.8	291.2	344.8	291.1	294.5
2015	29.7	94.0	147.0	201.8	219.0	255.4	277.3	300.0	304.3	312.3
2016	40.4	93.7	154.4	205.4	212.1	254.2	263.9	277.9	309.3	319.0
2017	33.2	95.2	149.8	174.5	225.4	220.3	271.9	265.5	294.9	305.5

Supplementary Table S6. The suicide by pesticide crude rates of the population age 15-64 by age, including workers and non-workers, per 1,000,000.

Year	Suicide by Pesticide Rate									
	**15-19**	**20-24**	**25-29**	**30-34**	**35-39**	**40-44**	**45-49**	**50-54**	**55-59**	**60-64**
2003	9.4	21.8	39.8	53.0	68.3	79.6	80.5	91.1	118.1	160.4
2004	5.8	19.1	26.4	37.6	58.6	79.1	90.5	108.9	142.2	181.6
2005	4.5	10.1	19.3	32.2	48.1	58.7	73.8	89.0	100.5	186.9
2006	2.6	5.5	11.2	19.3	33.6	45.8	66.1	84.1	104.5	140.3
2007	1.6	4.9	11.7	16.6	31.4	45.4	63.3	81.3	102.7	151.4
2008	2.6	6.9	11.6	17.9	34.6	45.3	59.7	56.9	84.9	104.9
2009	2.0	7.2	7.6	14.1	26.3	32.2	56.5	68.7	78.1	109.1
2010	0.9	2.0	7.3	16.8	22.2	31.5	53.0	57.1	73.7	105.9
2011	1.2	4.6	7.1	11.1	19.0	29.8	40.8	66.3	79.2	105.9
2012	2.0	1.6	2.3	8.1	12.2	21.8	32.4	50.3	60.0	87.5
2013	0.3	0.8	0.3	3.1	6.4	9.9	19.1	27.2	32.6	48.2
2014	0.3	0.8	0.6	3.1	3.6	7.1	12.5	21.1	22.5	34.0
2015	0.3	0.6	0.6	1.8	2.0	3.5	9.6	16.2	22.2	31.2
2016	0.3	0.3	1.2	1.0	2.3	4.6	8.7	11.7	24.0	33.0
2017	0.3	0.3	0.3	0.8	0.8	3.2	6.8	10.8	18.4	32.6

Supplementary Table S7. The suicide overall crude rates of the population age 15-64 by occupational groups per 1,000,000.

Year	MNP		OST		AFF		SKL		USL	
	Male	Female	Male	Female	Male	Female	Male	Female	Male	Female
2003	58.6	25	290.1	51.9	939	247	119.7	35.7	326	26.8
2004	67	40.6	312.1	48	1007.7	263.6	112.2	19.4	332.3	30.4
2005	88.9	40.7	305	69.9	957.8	257.7	104.3	24.2	281.6	30.3
2006	68	30.2	286.1	49.1	818.6	219.8	84.8	19.4	232.5	14
2007	76.2	68.1	293	85.8	851	208.8	84.2	48	193.3	37
2008	111.3	64.3	266.1	100.1	725.1	219.5	99.5	42.6	238	28.3
2009	183.8	97.1	354.2	124.4	742.4	212.1	119.6	41.9	307.3	26.5
2010	197.7	83.7	374.8	108.9	778.9	221.8	107.6	28.5	271.2	23.8
2011	179.1	77.4	377.3	99.7	807.9	212.9	123.9	25	293.8	21.2
2012	202.7	60.2	312.7	90.7	670.8	194	120.2	39.2	387.3	45.3
2013	261	72.6	315	90.5	702.9	153.5	141.5	32.8	484.1	50.7
2014	252.2	73	310.9	86.4	557.4	123.4	130.9	53.7	525	49.3
2015	247.8	56.7	276.4	87.6	618.6	266.7	133.6	44.6	481.1	37.1
2016	256.4	60.6	257.8	91.8	589.1	227.4	121.6	45	494	42.2
2017	244.7	52.4	246.9	82.8	530.5	292.8	113.3	26.3	409.4	42.4

MNP: Manager-Professional, **OST:** Officer-Service-Trade, **AFF:** Agriculture-Forestry-Fishery, **SKL:** Skilled manual labor, **USL:** Unskilled manual labor.

Supplementary Table S8. The suicide by pesticide crude rates of the population age 15-64 by occupational groups per 1,000,000.

Year	MNP		OST		AFF		SKL		USL	
	Male	Female	Male	Female	Male	Female	Male	Female	Male	Female
2003	9.9	6.6	69.6	22.2	554.2	154.1	29.9	11.9	107.7	10.7
2004	7.8	5.2	72.7	14.7	645.1	197.2	28.7	6.5	101.2	12.6
2005	11.9	2.4	51.3	9.7	581.7	183.1	22.5	4.4	63	7.3
2006	8.8	2.2	43	7.8	491.2	155.3	15.3	1.4	66.8	3
2007	9.8	0	43	7.1	555.7	140.9	15.8	3.4	42	9.7
2008	11.4	3.2	34.4	10.8	423.2	155.3	15.8	7.6	53	8.4
2009	19.2	4.1	33.4	9.1	435.8	146.2	15.2	3.4	56.9	9.6
2010	12.8	3	29.9	7.4	417.8	135	12.5	3.2	59	0.7
2011	15.9	3.4	28.5	7.9	419.2	151.6	12.1	6.3	51.6	4.2
2012	10.2	1.4	19.4	4.1	317.1	129.4	8.8	1.5	43.7	10.2
2013	9.2	1.3	9.5	2	251.2	73.4	6.9	1.5	36.4	2.9
2014	5.3	0.8	7.1	1.4	166.6	54.4	5.6	0	31.9	2.2
2015	4.8	0	5	2.5	135.5	64.6	5.5	1.5	20.6	0.7
2016	4.7	0	4.5	2.6	132.4	62	3.1	0	31.2	1.5
2017	3.7	1.2	4.5	2.2	82.8	97.6	3.1	0	17	2.3

MNP: Manager-Professional, **OST:** Officer-Service-Trade, **AFF:** Agriculture-Forestry-Fishery, **SKL:** Skilled manual labor, **USL:** Unskilled manual labor.

Supplementary Table S9. The suicide overall standardized mortality ratios of the population age 15-64 by occupational groups per 1,000,000.

Year	MNP		OST		AFF		SKL		USL	
	Male	Female	Male	Female	Male	Female	Male	Female	Male	Female
2003	16.9 (13.5-20.8)	24.8 (14.9-37.1)	92.1 (86.7-97.7)	48.9 (42.9-55.2)	206.2 (190.5-222.6)	198.6 (166.3-233.8)	37 (34.2-39.9)	33.8 (24.9-44)	89.3 (80.2-98.9)	22.9 (14.8-32.7)
2004	18.8 (15.2-22.8)	42.2 (28.6-58.3)	98.3 (92.6-104.1)	45.3 (39.6-51.4)	202.1 (186.4-218.3)	190.8 (159.1-225.4)	33.6 (31.1-36.3)	18.8 (12.4-26.6)	87 (78.4-96.1)	24.3 (16.3-34)
2005	24.9 (20.8-29.5)	29.7 (20.5-40.5)	94.8 (89.3-100.5)	52.1 (46.7-57.8)	194.7 (178.8-211.4)	173.1 (142.8-206.4)	30.7 (28.2-33.2)	18.1 (12.5-24.8)	72.2 (64.5-80.5)	22.2 (14.8-31)
2006	21.2 (17.3-25.6)	30.2 (19.9-42.6)	99.5 (93.5-105.8)	46.9 (41.1-53)	178.1 (161.9-195)	171.5 (138.2-208.3)	27.4 (25.1-29.9)	18.8 (12.5-26.4)	64.1 (56.6-72.2)	12.2 (6.6-19.3)
2007	22.8 (18.8-27.1)	38.6 (29.6-48.9)	96.3 (90.5-102.3)	53.8 (48.7-59.1)	187.7 (170.5-205.7)	140.2 (111.6-172)	25.8 (23.6-28.2)	29.7 (23.2-37.1)	52.4 (45.8-59.4)	25.1 (17.8-33.8)
2008	34.2 (30.6-38.1)	33.6 (27.9-39.9)	83.8 (78.8-88.9)	58.5 (53.4-63.8)	172.2 (155.2-190)	158 (125.7-194.1)	28.8 (26.1-31.7)	27.4 (18.2-38.5)	67.1 (59.8-74.9)	19.1 (13.5-25.8)
2009	44.8 (41.1-48.8)	44.1 (38.1-50.6)	88.7 (84.2-93.3)	62.8 (57.8-68)	141.2 (127.5-155.7)	125.1 (98.2-155.2)	27.3 (24.9-29.7)	23.1 (14.9-33)	69.6 (62.9-76.7)	15.2 (10.6-20.6)
2010	47.4 (43.7-51.3)	42.5 (36.3-49.1)	92.3 (87.8-97)	58.7 (53.8-63.9)	143 (129.3-157.3)	129.7 (100.9-162.1)	23.7 (21.6-26)	15.9 (9.4-24.1)	59.9 (53.8-66.3)	13.7 (9.4-18.8)
2011	40.4 (37-43.9)	44.5 (37.9-51.6)	87.8 (83.5-92.1)	58.7 (53.6-64.1)	138.1 (124.8-152)	122.1 (94.4-153.3)	25.5 (23.4-27.8)	14.9 (8.5-23)	60.8 (54.8-67)	12.5 (8.4-17.3)
2012	51.5 (47.4-55.7)	42.1 (35.1-49.6)	82.6 (78.3-87)	63.3 (57.6-69.3)	128.1 (114.6-142.3)	130.9 (99.1-167.1)	27.7 (25.3-30.1)	26.4 (17.2-37.5)	89 (81.3-97.1)	30.7 (23.5-38.8)
2013	67.4 (62.7-72.3)	50.2 (42.8-58.2)	84 (79.7-88.4)	64.9 (59-71)	151.4 (135.6-168.1)	118.3 (86.6-155)	33.7 (31.1-36.4)	23 (14.4-33.7)	120.3 (110.9-130.2)	37.6 (29.3-47.1)
2014	65.6 (61-70.3)	52.3 (44.8-60.4)	83.9 (79.6-88.3)	63.9 (58.1-70)	122.7 (108-138.3)	98.2 (68-134)	31.5 (29-34.1)	38.4 (26.9-52)	133.7 (123.8-144)	37.4 (28.9-46.9)
2015	70.6 (65.6-75.8)	47.5 (39.9-55.8)	82 (77.5-86.5)	74.6 (67.7-81.9)	135.2 (118.7-152.7)	224.8 (154.6-308)	34.3 (31.6-37)	37.1 (24.8-51.9)	128 (118.2-138.1)	30.9 (22.9-40.1)
2016	75.7 (70.5-81.2)	48.9 (41.3-57)	78.2 (73.9-82.7)	74.8 (68.1-81.9)	129.6 (113-147.4)	183.4 (114.8-268)	32.1 (29.5-34.9)	36 (24.1-50.3)	134.2 (123.9-144.8)	33.8 (25.5-43.3)
2017	74.1 (68.8-79.6)	45.2 (37.9-53.2)	76.6 (72.3-81.1)	72.1 (65.4-79.3)	121.1 (105.1-138.3)	247.4 (158.3-356.3)	30.9 (28.3-33.7)	21.8 (12.7-33.4)	115.1 (105.7-124.8)	35.6 (26.8-45.6)

The reference populations of 2005, 2010, and 2015 were used for each five-year period (2003-2007, 2008-2012, and 2013-2017). **MNP:** Manager-Professional, **OST:** Officer-Service-Trade, **AFF:** Agriculture-Forestry-Fishery, **SKL:** Skilled manual labor, USL: Unskilled manual labor.

Supplementary Table S10. The suicide by pesticide standardized mortality ratios of the population age 15-64 by occupational groups per 1,000,000.

Year	MNP		OST		AFF		SKL		USL	
	Male	Female	Male	Female	Male	Female	Male	Female	Male	Female
2003	9.5 (5.2-15.2)	17.8 (5.6-36.8)	76 (67.1-85.6)	52.6 (42.9-63.3)	358.9 (323.4-396.2)	275.7 (219.6-338.3)	31.5 (26.8-36.6)	28.7 (16.3-44.4)	92.9 (76.7-110.6)	21.1 (10-36.2)
2004	7.4 (3.7-12.3)	17.3 (4.5-38.4)	79.7 (70.3-89.6)	38.2 (29.8-47.7)	362.1 (327.2-398.7)	324.1 (262.2-392.6)	29.4 (25.1-34.2)	17.6 (8-31)	80.1 (66-95.5)	24.3 (12.5-40)
2005	13.7 (7.9-20.9)	10.7 (1-30.8)	68.2 (58.6-78.4)	32 (23.5-41.8)	384.2 (344.1-426.5)	324.6 (257.7-399.1)	27.7 (23.1-32.7)	15.5 (5.6-30.3)	58 (45.2-72.3)	16.7 (6.6-31.4)
2006	12.2 (6.5-19.7)	13.6 (1.3-39)	70 (59.3-81.6)	30.5 (21.5-40.9)	390 (344.6-438.2)	308.8 (238.3-388.4)	22.5 (18-27.4)	6 (0.6-17.1)	73.5 (57.8-91.1)	7.8 (1.5-19)
2007	13.5 (7.6-21.3)	0	69 (58.5-80.5)	28.6 (19.8-39)	417.8 (370.8-467.6)	319.2 (241.1-408.3)	22.8 (18.4-27.7)	15.1 (4.8-31.2)	44.1 (32.7-57.1)	27 (12.8-46.3)
2008	19.7 (13.5-27)	18.9 (6.8-37)	61.7 (51.8-72.3)	48 (35.8-61.9)	408.2 (356.1-464)	410.8 (311.9-523.4)	23.9 (18.4-30)	26.6 (8.4-55.1)	71 (55-89.1)	26 (12.9-43.7)
2009	36.8 (27.7-47.2)	27.4 (11.7-49.6)	66.4 (55.8-78)	43.8 (31.7-57.8)	425 (371.4-482.2)	391 (291.1-505.7)	24 (18.3-30.3)	12.4 (1.2-35.5)	78.6 (61.6-97.7)	30.4 (16.1-49.1)
2010	24.8 (17.5-33.3)	25.2 (9.1-49.4)	60.1 (49.9-71.2)	44.1 (30.7-60)	420.8 (366.3-479.1)	416.1 (299.8-551.5)	19.7 (14.7-25.4)	14.7 (1.4-42.2)	84.7 (66.8-104.6)	2.7 (0-10.7)
2011	34.4 (25.2-44.9)	23.7 (9.4-44.5)	64.8 (53.8-76.8)	40 (28.3-53.8)	420.3 (364.8-479.6)	425.8 (312.7-556.2)	20 (14.9-25.8)	24.9 (6.5-55.4)	75.5 (58.6-94.6)	14.1 (5.1-27.7)
2012	29 (19.5-40.3)	13.8 (2.6-33.9)	58.9 (47.1-72.1)	27.2 (16.6-40.5)	403.3 (342.5-469.1)	451.8 (319.6-606.9)	18.7 (13.2-25.2)	7.7 (0-30.1)	80.6 (60.7-103.4)	42.4 (23.1-67.5)
2013	47.9 (31.5-67.7)	27.3 (5.2-67)	51.3 (37.1-67.7)	27.5 (13.1-47.1)	588.1 (487.2-698.6)	459.1 (287.3-671)	25.9 (17.5-36)	15.3 (0-60.1)	123.2 (89.8-161.8)	22.7 (5.9-50.4)
2014	37.3 (21.3-57.8)	24 (2.3-68.8)	50.6 (34.8-69.4)	27.1 (10.8-51)	507.8 (399.4-629.2)	609.4 (340-956.8)	27.8 (18-39.8)	0	145.7 (104.5-193.7)	29 (5.5-71.1)
2015	42 (22.9-66.9)	0	45 (28.5-65.2)	62.5 (32.1-102.8)	443.3 (332-570.6)	814.1 (347.7-1476)	32.4 (20.9-46.3)	29.5 (0-115.8)	106.9 (70.4-151)	11.2 (0-43.9)
2016	43.5 (23.7-69.3)	0	42.5 (26.3-62.7)	59.8 (31.7-96.7)	437 (323.2-568)	623.4 (224.4-1222.1)	18.7 (10.2-29.8)	0	168.8 (120.6-225.2)	18.6 (1.8-53.4)
2017	43.5 (21.6-73)	46.7 (8.8-114.5)	52.1 (32.2-76.7)	51.4 (25.5-86.3)	322.7 (220.6-444.2)	870 (371.6-1577.4)	23.4 (12.8-37.3)	0	112.1 (70.9-162.5)	26.6 (5-65.3)

[1]The reference populations of 2005, 2010, and 2015 were used for each five-year period (2003-2007, 2008-2012, and 2013-2017). **MNP:** Manager-Professional, **OST:** Officer-Service-Trade, **AFF:** Agriculture-Forestry-Fishery, **SKL:** Skilled manual labor, USL: Unskilled manual labor.

Supplementary Table S11. The time trends of three major economic indicators in percentage (%), which are the real gross domestic product (RGDP), the unemployment rate (UnR), and the rate of customers price index (CPI) from one year to the next.

Year	RGDP	UnR	CPI
2003	3.1	3.7	3.5
2004	5.2	3.8	3.6
2005	4.3	3.9	2.8
2006	5.3	3.6	2.2
2007	5.8	3.4	2.5
2008	3	3.3	4.7
2009	0.8	3.8	2.8
2010	6.8	3.8	2.9
2011	3.7	3.5	4
2012	2.4	3.3	2.2
2013	3.2	3.2	1.3
2014	3.2	3.6	1.3
2015	2.8	3.7	0.7
2016	2.9	3.8	1
2017	3.2	3.8	1.9

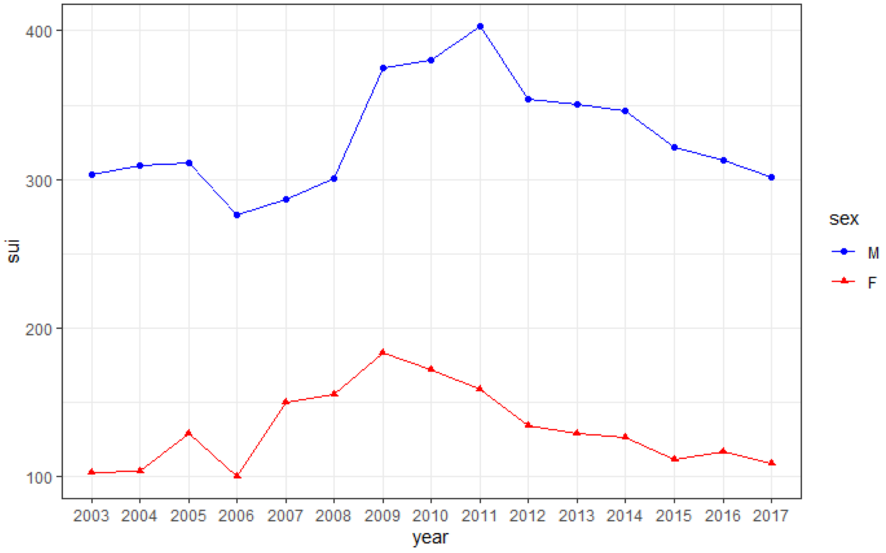

(a) Crude rates of overall suicide by sex

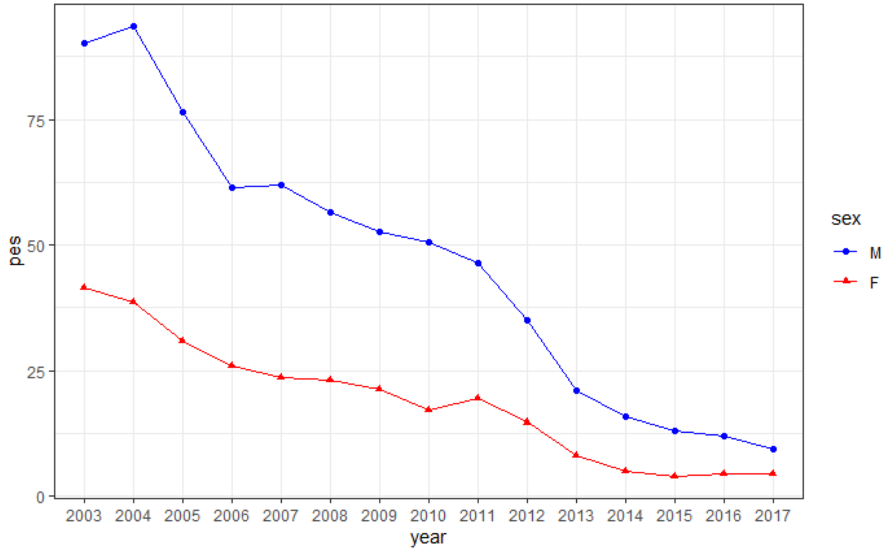

(b) Crude rates of suicide by pesticide by sex

Supplementary Figure 1. The overall suicide and suicide by pesticide rates by sex of the population age 15-64, including workers and non-workers, per 1,000,000.

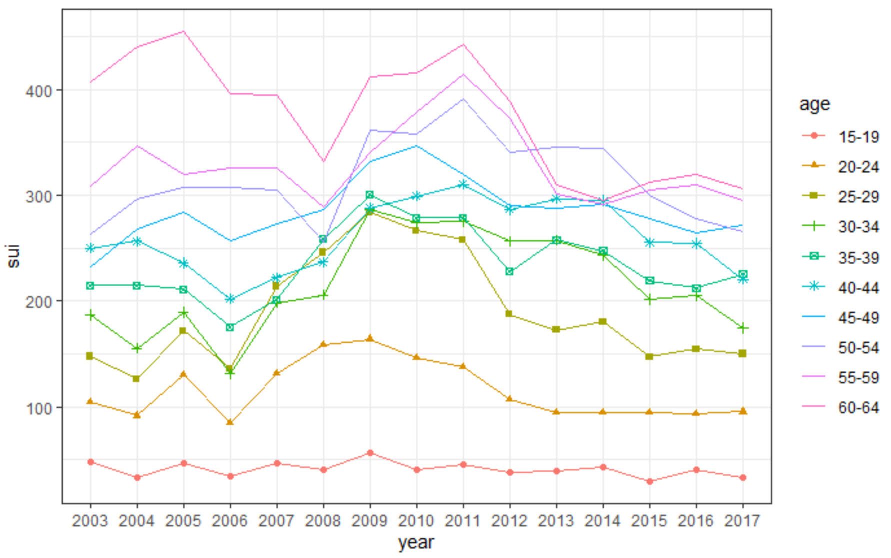

(a) Crude rates of suicide overall by age

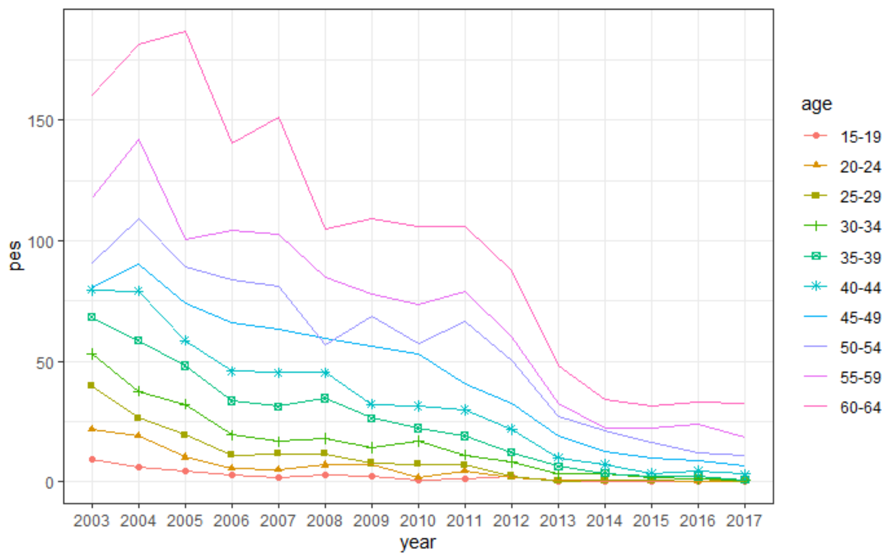

(b) Crude rates of suicide by pesticide by age

Supplementary Figure 2. The suicide overall and suicide by pesticide rates by age of the population age 15-64, including workers and non-workers, per 1,000,000.
